# 2017 Sowing the “CEED”s of a more diverse biomedical workforce

**DOI:** 10.1017/cts.2018.226

**Published:** 2018-11-21

**Authors:** Colleen A. Mayowski, Kaleab Z. Abebe, Natalia E. Morone, Doris M. Rubio, Wishwa N. Kapoor

**Affiliations:** 1 University of Pittsburgh; 2 Institute for Clinical Research Education, University of Pittsburgh School of Medicine

## Abstract

OBJECTIVES/SPECIFIC AIMS: The need to diversify the biomedical research workforce is well documented. The Career Education and Enhancement for Health Care Research Diversity (CEED) program at the University of Pittsburgh Institute for Clinical Research Education (ICRE) promotes success and helps seal the “leaky pipeline” for under-represented background (URB) biomedical researchers with a purposefully designed program consisting of a monthly seminar series, multilevel mentoring, targeted coursework, and networking. METHODS/STUDY POPULATION: Over 10 program years, we collected survey data on characteristics of CEED Scholars, such as race, ethnicity, and current position. We created a matched set of URB trainees not enrolled in CEED during that time using propensity score matching in a 1:1 ratio. RESULTS/ANTICIPATED RESULTS: Since 2007, CEED has graduated 45 Scholars. Seventy-six percent have been women, 78% have been non-White, and 33% have been Hispanic/Latino. Scholars include 20 M.D.s and 25 Ph.D.s. Twenty-eight CEED Scholars were matched to non-CEED URB students. Compared with matched URB students, CEED graduates had a higher mean number of peer-reviewed publications (9.25 vs. 5.89; *p*<0.0001) were more likely to hold an assistant professor position (54% vs. 14%; *p*=0.004) and be in the tenure stream (32% vs. 7%; *p*=0.04), respectively. There were no differences in Career Development Awards (*p*=0.42) or Research Project Grants (*p*=0.24). DISCUSSION/SIGNIFICANCE OF IMPACT: Programs that support URB researchers can help expand and diversify the biomedical research workforce. CEED has been successful despite the challenges of a small demographic pool. Further efforts are needed to assist URB researchers to obtain grant awards.

